# Visual Predictors of Postural Sway in Older Adults

**DOI:** 10.1167/tvst.11.8.24

**Published:** 2022-08-24

**Authors:** Joanne M. Wood, Callula Killingly, David B. Elliott, Kaarin J. Anstey, Alex A. Black

**Affiliations:** 1 Centre for Vision and Eye Research, School of Optometry and Vision Science, Queensland University of Technology, Brisbane, Australia; 2 School of Early Childhood and Inclusive Education, Queensland University of Technology, Brisbane, Australia; 3 School of Optometry and Vision Science, University of Bradford, Bradford, UK; 4 School of Psychology, University of New South Wales, Sydney, Australia; 5 Neuroscience Research Australia, Sydney, Australia; 6 UNSW Ageing Futures Institute, University of New South Wales, Sydney, Australia

**Keywords:** postural sway, older adults, eye disease, vision impairment, motion sensitivity, visual fields

## Abstract

**Purpose:**

Accurate perception of body position relative to the environment through visual cues provides sensory input to the control of postural stability. This study explored which vision measures are most important for control of postural sway in older adults with a range of visual characteristics.

**Methods:**

Participants included 421 older adults (mean age = 72.6 ± 6.1), 220 with vision impairment associated with a range of eye diseases and 201 with normal vision. Participants completed a series of vision, cognitive, and physical function tests. Postural sway was measured using an electronic forceplate (HUR Labs) on a foam surface with eyes open. Linear regression analysis identified the strongest visual predictors of postural sway, controlling for potential confounding factors, including cognitive and physical function.

**Results:**

In univariate regression models, unadjusted and adjusted for age, all of the vision tests were significantly associated with postural sway (*P* < 0.05), with the strongest predictor being visual motion sensitivity (standardized regression coefficient, β = 0.340; age-adjusted β = 0.253). In multiple regression models, motion sensitivity (β = 0.187), integrated binocular visual fields (β = −0.109), and age (β = 0.234) were the only significant visual predictors of sway, adjusted for confounding factors, explaining 23% of the variance in postural sway.

**Conclusions:**

Of the vision tests, visual motion perception and binocular visual fields were most strongly associated with postural stability in older adults with and without vision impairment.

**Translational Relevance:**

Findings provide insight into the visual contributions to postural stability in older adults and have implications for falls risk assessment.

## Introduction

Balance or postural stability is controlled through input from the visual, vestibular, and somatosensory systems. Of these, the visual system provides one of the most important sources of information.^1^ The visual contribution to postural stability also increases with age, in order to compensate for age-related deteriorations in the somatosensory and vestibular systems.^2^ Importantly, if there is failure to adapt to the increasing reliance on vision with aging, postural stability is reduced,^3^^,^^4^ which can lead to an increased risk of falls.^5^^–^^7^

Several vision measures have been reported to be associated with postural stability in older adults, particularly visual acuity, visual fields, and contrast sensitivity. Reduced visual acuity induced by defocus has been shown to reduce postural stability, particularly when the vestibular and somatosensory systems are disrupted, in both young^1^^,^^8^ and older^9^^,^^10^ adults, and correction of myopic refractive errors has been shown to improve postural stability.^11^ Similarly, degrading vision through simulated cataracts also reduces postural stability.^10^ Older individuals with moderately impaired visual acuity from a range of age-related eye diseases (worse than 20/60) also demonstrated poorer postural stability (assessed using the Berg balance scale) compared to those with normal vision or mild vision impairment (20/60 or better).^12^ Older adults with reductions in contrast sensitivity and stereopsis have reduced postural stability, and these visual functions were shown to be independent predictors of postural stability while standing on foam (which disrupts the somatosensory system).^13^ Similarly, contrast sensitivity was the only vision measure significantly associated with sway on a foam surface in older adults with age-related macular degeneration (AMD), when the visual function measures were combined in a multivariate model.^14^ This is also consistent with another study that reported a significant association between contrast sensitivity and postural sway on foam in adults with AMD, but little association between postural sway and central visual field measures.^15^

Visual field loss has also been shown to play a role in postural stability but with mixed results. Binocular visual field loss derived from integrating monocular suprathreshold fields (Humphrey Field Analyser [HFA] 81-point single intensity screening) was associated with increased postural sway in individuals with glaucoma, both on a firm and foam surface, independent of age, gender, body mass index, and physical performance levels.^16^ Furthermore, those individuals with glaucoma and greater inferior integrated field loss showed increased postural sway on the foam surface,^16^ which was supported by the findings of a study of older adults with AMD, in whom inferior integrated field loss (HFA monocular 24-2 fields combined) was associated with greater postural sway on foam than superior field loss.^14^ However, in another study of individuals with glaucoma and age-similar controls, binocular field defects were not found to be associated with postural sway but were associated with the relative visual and somatosensory contribution to sway in this population.^17^

Motion perception has also been shown to play a role in postural stability.^18^ During quiet stance, the body exhibits small oscillatory movements that result in optic flow cues in the retina, which provide input to the control of postural stability.^19^^,^^20^ The role of optic flow across the visual field on postural stability has also been highlighted in experimental studies that used the moving room paradigm, where older adults demonstrated greater postural sway than younger adults.^21^^,^^22^ However, despite the association between motion cues and postural sway being demonstrated in experimental laboratory-based studies, few studies have explored the association between motion perception and balance in older adults in large cross-sectional population studies. Turano et al.,^19^ in a small study of older adults with and without AMD, reported that minimum displacement thresholds (D_min_) using random dot kinematograms were the strongest predictors of sway rather than visual acuity, contrast sensitivity, or visual fields and concluded that self-motion cues generated by small body oscillations are unlikely to be detected in those with impaired D_min_, leading to increased sway. In a large population-based cohort, D_min_ was reported to be the strongest predictor of the ability to complete a series of timed stands of increasing difficulty in older adults, compared to visual acuity, contrast sensitivity, and visual fields; however, postural sway was not measured using a standardized force plate.^23^

In this study, we expanded on previous studies to explore the association of a range of visual function measures with postural sway as measured using an electronic force plate in older adults. We included participants with and without eye disease, to provide a wide range of visual characteristics, and adjusted for confounding factors.

## Methods

### Participants

Participants were community-dwelling older adults aged 60 years and above, with or without eye disease, and were current drivers, as they had been participants in various older driver studies. Exclusion criteria included Parkinson's disease, a history of dizziness or vestibular disease, use of a walking aid, or cognitive impairment (Mini-Mental State Examination score <24 of 30).^24^ Participants with eye disease were recruited from the clinical records of the Queensland University of Technology (QUT) optometry clinic and local private ophthalmology practices. The age-similar participants with normal vision and without eye disease were recruited from our existing database of volunteers with no eye disease and from the QUT optometry clinic and newspaper advertisements.

The study followed the tenets of the Declaration of Helsinki and was approved by the QUT Human Research Ethics Committee. Participants were given a full explanation of the study, experimental procedures, and possible consequences, and written informed consent was obtained before participant assessment. All testing was conducted in the same session.

### Visual Function Assessment

Participants underwent a comprehensive eye examination conducted by an experienced optometrist, including indirect ophthalmoscopy, slit-lamp biomicroscopy, and fundus photography, to identify the presence or absence of eye disease. Participants then completed a battery of visual tests while wearing their habitual distance correction.

#### Visual Acuity

Distance high-contrast visual acuity was measured binocularly with the Early Treatment for Diabetic Retinopathy Study chart at 5 m and a luminance of 100 cd/m^2^, using the letter-by-letter scoring method.^25^

#### Contrast Sensitivity

Letter contrast sensitivity was measured binocularly using the Pelli-Robson Contrast Sensitivity chart at 1 m and a luminance of 110 cd/m^2^, using the letter-by-letter scoring method. ^26^ A +1.00 DS lens was used to compensate for the working distance.

#### Visual Fields

Monocular visual fields were assessed using the SITA-Standard 24-2 threshold strategy on a HFA (model 750; Carl Zeiss-Meditec, Dublin, CA, USA). The monocular visual fields were combined to form an integrated binocular field based on the more sensitive of the two eyes at each location.^27^

#### Motion Sensitivity (D_min_)

Central motion sensitivity was measured binocularly using a computer-generated random-dot kinematogram at 3 m.^28^^,^^29^ A square patch of dots was presented (5.1° × 5.1°) within which a smaller patch of dots (4.1° × 4.1°) moved in one of four directions (up, down, left, or right providing a four-alternative forced-choice task) over four discrete steps of 150 ms. Participants reported the direction in which the central patch of dots was perceived to be moving. Pixel displacement between frames was varied in a two-down one-up staircase, with eight reversals; thresholds were the minimum displacement threshold (log degree arc).

### Assessment of Confounding Factors

Cognitive and physical measures were included as potential confounding factors as well as the weight and height of participants, given that both impaired cognitive ability and poor physical function have been reported to be associated with reduced postural stability^30^^,^^31^ and vision impairment.^32^^,^^33^

#### Anthropometric Measures

Height and weight were recorded using standard scales.

#### Trails A and B

Cognitive motor speed and task-switching ability, an aspect of executive function, were measured with pen-and-paper versions of the Trails A and B tests.^34^ Participants were instructed to draw lines to connect circles containing either a sequence of 25 numbers (1–2–3 … 25; TMA) or to alternate between a total of 25 numbers and letters (i.e., 1–A–2–B … L–13; TMB) in sequence as quickly as they could without making mistakes. Performance was recorded as the total time taken to complete the test.

#### Digit Symbol Substitution

Processing speed, short-term memory, and attention switching were measured using a paper-based version of the Digit Symbol Substitution Test.^35^ Participants identified which symbol corresponded with a number derived from a coding key and the score given as the number of symbols correctly coded within 90 seconds.

#### Color Choice Reaction Time

A computer-based test of reaction times that assesses attention, vigilance, divided attention, and response inhibition was administered.^29^^,^^36^ Images of red and blue cars were presented at random intervals in one of four quadrants of the computer screen, and participants responded when a car appeared, either with their hands (left or right hand for the corresponding quadrants in the upper quadrants) or feet (left and right feet [via a foot pedal] for the corresponding lower quadrants). Participants were instructed not to respond to blue cars to measure inhibition. Average reaction time for correct trials and the total number of correct trials were recorded.

#### Timed Up and Go Test

The timed up and go (TUG) test is a well-established test of lower extremity and mobility,^37^ which includes some level of executive cognitive function.^38^^,^^39^ Participants were instructed to rise from a seated position from a 46-cm high chair, walk 3 m at their usual pace, and then turn around and walk back to the chair and sit down. The time taken from the instruction to stand up to when the participant's back was resting against the chair was recorded in seconds using a digital stopwatch. Participants were given a practice run and then completed two trials, with the mean value for the two trials calculated.

#### Handgrip Strength

Grip strength has been shown to provide an overall indication of general frailty, being closely associated with and predictive of future mobility.^40^ To assess handgrip strength (in kilograms of force), participants squeezed a portable dynamometer (North Coast Medical Inc., Morgan Hill, CA, USA) as hard as possible with their elbow flexed at right angles to the forearm; three measurements were taken for each hand and the mean value calculated and represented as grip strength in the better and worse hand.

#### Walking Speed

Walking speed was assessed as it has been shown to provide a useful indication of physical function and disability in older adults.^41^^,^^42^ Participants walked twice, back and forth along a 23-m-long indoor corridor, free of obstacles, at their preferred normal walking speed; the total time taken was recorded using a digital stopwatch, and walking speed (in m/s) was calculated and averaged across the two trials.

### Postural Sway Assessment

Postural sway was assessed using an electronic Force Platform (HUR Labs, Tampere, Finland) on a foam surface (medium-density foam block, 80 × 70 × 25 cm), to reduce somatosensory feedback, with eyes open. Participants stood on the force plate with bare feet in a standardized stance position, approximately 17 cm apart between the inner edges of the heels and feet abducted to 14 degrees,^43^ keeping as still as possible with their arms relaxed at their sides, as in normal quiet stance. Participants wore their habitual distance correction (if any) and were instructed to look straight ahead at a large high-contrast distance fixation target (15-cm × 15-cm cross at 6 m) during the test. Postural sway was assessed for 30 seconds with a sampling rate of 50 Hz using the center of pressure (CoP) signal recorded by the force platform and represented by the total length of the CoP path in millimeters.^8^^–^^10^^,^^14^^,^^44^

### Statistical Analysis

Statistical analyses were performed using SPSS statistical software v25.0 (SPSS, Inc., Chicago, IL, USA), and the level of significance was set at *P* < 0.05. Independent *t*-tests were used to compare differences in visual function between the eye disease and normal vision groups. A series of univariate regression analyses was conducted to determine the association between the vision function measures and postural sway on foam with eyes open; all associations were also adjusted for age. Multiple regression models were then conducted to determine the best visual predictors of sway, adjusted for potential confounders. In the initial regression model, all the vision variables and age were included in a backward stepwise model, with *P* = 0.10 used as the significance level for removal. Next, confounding was assessed on the association between the retained vision measures and postural sway for all of the cognitive and physical measures, as well as gender, weight, and height; confounders were assessed in separate models. A final model was constructed that included age and the vision variables retained in the initial model, plus the identified confounders. Given the likely correlations between some of the vision measures, multicollinearity was checked in each of the models by calculating the variance inflation factor.

## Results

Participants included 421 older adults (mean age 72.6 ± 6.1 years; 144 female), of whom 220 had vision impairment arising from a range of eye diseases, including cataract, glaucoma, and age-related macular degeneration, and 201 had no significant eye disease and normal vision. Overall, the length of the CoP path for all participants was 666.1 ± 267.1 mm, with the eye disease group demonstrating significantly greater sway than those without eye disease (713.4 ± 280.8 vs. 614.4 ± 241.6; *t*_419_ = 5.15; *P* < 0.001). As expected, visual function was significantly poorer for all vision measures for the group with eye disease (all *P* < 0.001).

Table 1 presents the visual function characteristics of all participants and the univariate associations with postural sway, unadjusted and adjusted for age. All of the vision tests were significantly associated with postural sway (*P* < 0.05), with motion sensitivity having the strongest association (standardized regression coefficient, β = 0.340), where reduced motion sensitivity was associated with greater postural sway.

**Table 1.** Visual Function Characteristics of All Participants (*N* = 421) and Univariate Associations With Postural Sway (Eyes Open on Foam), Unadjusted and Adjusted for Age

Visual Function	Mean (SD)	Range	Crude Standardized Regression Coefficient (β)	*P*-Value	Age-Adjusted Standardized Regression Coefficient (β)	*P*-Value
Binocular visual acuity (logMAR)	−0.02 (0.11)	−0.24 to 0.42	0.235	<0.001	0.132	0.005
Binocular contrast sensitivity (logCS)	1.76 (0.11)	1.15 to 1.90	−0.245	<0.001	−0.164	<0.001
Monocular 24-2 MD better eye (dB)	−0.19 (3.05)	−23.24 to 4.25	−0.199	<0.001	−0.163	<0.001
Monocular 24-2 MD worse eye (dB)	−2.75 (5.79)	−31.00 to 3.54	−0.216	<0.001	−0.185	<0.001
Integrated binocular field central 20 deg (dB)	30.65 (2.28)	19.7 to 44.40	−0.259	<0.001	−0.158	0.001
Motion sensitivity (logdegarc)	−1.68 (0.25)	−2.04 to −0.82	0.340	<0.001	0.253	<0.001

dB, decibels; logCS, logarithm of contrast sensitivity; logdegarc= logarithm of degrees of arc; logMAR, logarithm of the minimum angle of resolution; MD, mean deviation.

**Table 2.** Results of the Multiple Regression Models of Visual Predictors of Postural Sway (Eyes Open on Foam)

	Initial Model^a^		Final Model^b^	
Characteristic	Standardized Regression Coefficient β	*P*-Value	Standardized Regression Coefficient β	*P*-Value
Motion sensitivity	0.205	<0.001	0.187	<0.001
Integrated visual field	−0.123	0.008	−0.109	0.02
Age	0.281	<0.001	0.234	<0.001
Trails A			0.084	0.08
TUG test			0.104	0.08
Walking speed			0.026	0.65
Model fit	*F*_3, 417_ = 37.32; *R*^2^ = 21.2	<0.001	*F*_6, 414_ = 20.14; *R*^2^ = 22.6	<0.001

a Initial model—backward stepwise entry including all vision measures plus age (*P* = 0.10 used as the significance level for removal).

b Final model—initial model variables plus confounders.

The initial backward stepwise regression model included all the vision measures and age, with motion sensitivity, integrated visual fields, and age retained; this model explained 21% variance in postural sway, *F*_3, 417_ = 37.32, *P* < 0.001, *R*^2^ = 0.21 (Table 2). Variance inflation values were all below 1.21, indicating no multicollinearity concerns. Several confounding factors were identified from the cognitive and physical function measures, gender, weight, and height, where the difference between the crude and unadjusted association measures was >10%; the identified confounders included Trails A, TUG, and walking speed. The final model, which included the vision variables and age from the initial model, plus the identified confounders, explained 23% variance in postural sway, *F*_6, 414_ = 20.14, *P* < 0.001, *R*^2^ = 0.23, with the same variables being significant as in the initial model. Variance inflation values were all below 1.82, indicating no multicollinearity concerns.

## Discussion

In this study, the role of visual function in the control of postural sway was explored in a large group of older adults who had either normal vision or vision impairment from a range of eye diseases. Of the visual function measures included in this study, motion perception and integrated binocular visual fields, rather than visual acuity and contrast sensitivity, were most strongly associated with postural sway on foam, even after adjustment for age and confounding cognitive and physical function factors. Of the vision measures in the final model, motion sensitivity was most strongly associated with postural sway and greater than the other measures of visual function included in this study.

The finding that motion perception was most strongly associated with postural sway supports previous studies that involved participants with AMD, as well as general populations of community-dwelling adults,^19^^,^^23^ and extends these findings for a large population of older adults both with and without vision impairment. Importantly, the association of motion sensitivity with postural sway was evident even when adjusted for potential confounding factors. The role of motion perception in postural control, as revealed in this and other studies, is highly relevant, given that age-related decreases in motion perception can occur in both central and peripheral vision, as well as under mesopic and photopic light levels.^45^^,^^46^ Motion perception has also been shown to be impaired in a number of age-related eye diseases, including glaucoma^47^^,^^48^ and AMD.^49^ However, whether changes in motion perception are related to the increase in the rate of falls reported in older adults with these eye diseases^50^^–^^54^ has not been explored and needs further investigation.

The finding that a measure of central motion sensitivity was associated with postural sway is interesting given that the entire visual field is likely to contribute to the detection of motion and therefore maintenance of postural stability, as demonstrated by the role of optic flow in maintaining postural stability.^21^^,^^22^ However, although the current study only used a central measure of D_min_ using random dot kinematograms, it has been demonstrated that motion perception in the periphery can be predicted from central motion tests for these measures of motion sensitivity.^45^ The D_min_ measure of motion sensitivity also relies to some degree on central visual functions such as visual acuity and contrast sensitivity. Thus, in the present study, D_min_ is likely to have assessed both motion and other central vision function measures concurrently.

The finding that a measure of binocular integrated visual field loss was also associated with increased postural sway in the final adjusted models supports previous studies of older adults with eye diseases, including glaucoma and AMD.^14^^,^^16^ The fact that visual field loss is associated with postural stability is in accord with reports of the importance of detection of optic flow in the peripheral field,^22^^,^^55^ suggesting that both central and peripheral visual cues are important for the maintenance of balance in older adults.

The findings of this study need to be considered in light of its strengths and weaknesses. Strengths include a relatively large sample of older adults who were community dwelling with a wide range of visual characteristics and assessment of a wider range of visual functions and confounding factors (cognitive and physical function measures, age, weight, height, and gender) than previous studies. Another strength is the use of an electronic force plate measure of balance involving commonly used assessment protocols; however, the findings are based on a single 30-second static measure of balance, rather than using multiple measures. The weight of the participants may also have influenced the compression of the foam used to assess postural sway,^56^ which is why this characteristic was explored as a potential confounding factor in the models. There was also a likely recruitment bias toward more highly functioning older adults, as they were all current drivers; therefore, the range of visual, cognitive, and physical functions tended to include better function (e.g., binocular visual acuities were 20/40 or better in all but two participants who held conditional licenses and only ∼10% of participants had TUG scores that were worse than average for age^57^). It is also important to highlight that the purpose of this study was not to develop a comprehensive model of functional predictors of postural sway, given that the focus was to explore visual predictors of sway.

The findings of this study highlight the strong contribution of motion perception in the control of postural sway, more so than contrast sensitivity, visual acuity, and visual fields, which are typically reported to be associated with postural stability. Since increased postural sway is associated with increased falls risk in older adults,^5^^–^^7^ the findings of the present study provide further understanding of the impact of vision impairment, particularly impaired motion perception, on balance control and potential risk of falls in older adults.
